# Expert consensus on regenerative endodontic procedures

**DOI:** 10.1038/s41368-022-00206-z

**Published:** 2022-12-01

**Authors:** Xi Wei, Maobin Yang, Lin Yue, Dingming Huang, Xuedong Zhou, Xiaoyan Wang, Qi Zhang, Lihong Qiu, Zhengwei Huang, Hanguo Wang, Liuyan Meng, Hong Li, Wenxia Chen, Xiaoying Zou, Junqi Ling

**Affiliations:** 1 Department of Operative Dentistry and Endodontics, Hospital of Stomatology, Guanghua School of Stomatology, Sun Yat-Sen University & Guangdong Provincial Key Laboratory of Stomatology, Guangzhou, Guangdong China; 2 Department of Endodontology, Maurice H Kornberg School of Dentistry, Temple University, Philadelphia, Pennsylvania USA; 3 Department of Cariology and Endodontology, Peking University School and Hospital of Stomatology & National Center of Stomatology & National Clinical Research Center for Oral Diseases & National Engineering Laboratory for Digital and Material Technology of Stomatology & Beijing Key Laboratory of Digital Stomatology & Research Center of Engineering and Technology for Computerized Dentistry Ministry of Health & NMPA Key Laboratory for Dental Materials, Beijing, China; 4 State Key Laboratory of Oral Diseases & Department of Operative Dentistry and Endodontics, West China Hospital of Stomatology, National Center of Stomatology, National Clinical Research Centre for Oral Diseases, Sichuan University, Chengdu, China; 5 Department of Endodontics, Stomatological Hospital and Dental School of Tongji University, Shanghai Engineering Research Center of Tooth Restoration and Regeneration, Shanghai, China; 6 Department of Endodontics, School of Stomatology, China Medical University, Shenyang, Liaoning China; 7 Department of Endodontics, Shanghai Ninth People’s Hospital, Shanghai Jiao Tong University School of Medicine; College of Stomatology, Shanghai Jiao Tong University; National Clinical Research Center for Oral Diseases; National Center for Stomatology; Shanghai Key Laboratory of Stomatology, Shanghai, China; 8 Department of Operative Dentistry & Endodontics, School of Stomatology, The Fourth Military Medical University, Xi’an, China; 9 The State Key Laboratory Breeding Base of Basic Science of Stomatology (Hubei-MOST) & Key Laboratory of Oral Biomedicine Ministry of Education, School and Hospital of Stomatology, Wuhan University, Wuhan, China; 10 Department of Endodontics, Beijing Stomatological Hospital, School of Stomatology, Capital Medical University, Beijing, China; 11 College of Stomatology, Hospital of Stomatology, Guangxi Medical University, Nanning, Guangxi China

**Keywords:** Dentistry, Endodontics

## Abstract

Regenerative endodontic procedures (REPs) is a biologic-based treatment modality for immature permanent teeth diagnosed with pulp necrosis. The ultimate objective of REPs is to regenerate the pulp-dentin complex, extend the tooth longevity and restore the normal function. Scientific evidence has demonstrated the efficacy of REPs in promotion of root development through case reports, case series, cohort studies, and randomized controlled studies. However, variations in clinical protocols for REPs exist due to the empirical nature of the original protocols and rapid advancements in the research field of regenerative endodontics. The heterogeneity in protocols may cause confusion among dental practitioners, thus guidelines and considerations of REPs should be explicated. This expert consensus mainly discusses the biological foundation, the available clinical protocols and current status of REPs in treating immature teeth with pulp necrosis, as well as the main complications of this treatment, aiming at refining the clinical management of REPs in accordance with the progress of basic researches and clinical studies, suggesting REPs may become a more consistently evidence-based option in dental treatment.

## Introduction

Pulpal and periradicular pathosis, which can be caused by caries, trauma, or dental abnormalities, is a common disease in clinic for dental treatment. Root canal treatment (RCT) is a conventional option to manage the endodontic diseases in fully developed permanent teeth demonstrating outstanding clinical outcomes, while apexification procedure is the traditional treatment modality for the immature permanent teeth with pulp necrosis.^1,2^ The purpose of endodontic treatment is to eliminate pulp and periapical inflammation/infection and preserve the teeth. However, these treatment procedures remove the pulp and some dentin tissues, which weaken the dentinal strength, immunological responses and proprioceptive functions, leading to a higher risk of reinfection and tooth fracture.^3,4^ How to reconstruct vital pulp and restore the biological function of teeth becomes the goal of contemporary endodontics.

The concept of pulp tissue regeneration was first raised by Nygaard-Østby in the 1960s,^5^ laying the foundation of regenerative endodontics. In 2001, Iwaya et al.^6^ used revascularization to successfully induce root development and regain pulpal sensitivity in a young permanent tooth with apical periodontitis. In 2004, Banchs and Trope^7^ reported a case with a modified protocol of revascularization, specifically creating a blood clot in the canals after disinfection as a matrix for new tissue growth and a bacterial-tight coronal seal to prevent bacterial invasion into the pulp space, providing evidence for clinical application of regenerative endodontics.

Currently, strategies of regenerative endodontics mainly involve stem cell transplantation, cell homing and regenerative endodontic procedures (REPs).^8–10^ Stem cell transplantation was reported to successfully regenerate the pulp tissue in clinic, while only REPs has been widely applied in clinical practice. REPs is a biologically based approach incorporating the concept of tissue engineering to replace damaged structures including dentin and cells of the pulp-dentin complex,^8^ aiming at not only apical lesion healing, resolution of signs and symptoms, but also continued root development and strengthened dentinal tissues to prevent potential root fracture.^11,12^ The ultimate goal is to regenerate a functional pulp-dentin complex and regain the immune competency and normal nociception.

Since the first case reported in 2001, there has been an increase in studies using REPs to treat young permanent teeth with apical periodontitis.^7,12–16^ To date, more than 600 articles are found at PubMed when searching with the keywords “revascularization”, “revitalization” and “regenerative endodontics”.^17^ Article types include basic researches, case reports, case series, retrospective studies, prospective clinical trials, reviews and guidelines, the majority of which are case reports, basic researches and reviews. At present, two guidelines on REPs are available for dental practitioners. The American Association of Endodontists (AAE) published the clinical guideline on REPs in 2013, and updated the versions in 2016, 2018 and 2021.^18^ The European Society of Endodontology (ESE) position statement published in 2016^19^ provides similar procedure details but a few differences in medicament choice, blood clot formation, placement of capping materials and success criteria of REPs.

Terminologies in the REPs field have been evolving, including pulp revascularization,^7,20^ revitalization,^21,22^ regenerative endodontic therapy^23,24^ and regenerative endodontic procedures.^25,26^ Among them, the term “pulp revascularization” has been most commonly used in the publications. However, it should be emphasized that the regenerative procedures aim at not only reestablishment of vascularity in pulp tissues but also functional regeneration of pulp-dentin complex. From this perspective, it is inaccurate to simply name the regenerative endodontic modality as “pulp revascularization”. In 2007, Murray et al.^8^ introduced the term of regenerative endodontic procedures to recapitulate the tissue engineering essence of pulp regeneration. The AAE^18^ adopted this terminology in its clinical guideline and also included it in the Glossary of Endodontic Terms of 2020, while ESE^19^ referred to this biologically based approach as revitalization. In this expert consensus, “regenerative endodontic procedures” is used to describe this strategy.

## Biological foundation and histological evaluation of REPs

### Biological foundation of REPs

Over the last several decades, the advancement in tissue engineering has profoundly reshaped our thinking in medical treatment and reinvigorated regenerative medicine. Researches in dental pulp tissue engineering are supporting and laying a solid biological foundation for the development and promotion of REPs, which involves three key elements: stem cells, growth factors, and scaffolds.^27^

#### Stem cells

Till now, different populations of adult stem cells have been identified and can be induced to differentiate into odontoblast-like cells in specific conditions, showing their potentials to be used in REPs. These include dental pulp stem cells (DPSCs), stem cells of the apical papilla (SCAPs), periodontal ligament stem cells (PDLSCs), inflammatory periapical progenitor cells (iPAPCs) and bone marrow stem cells (BMSCs).^12^

Even when teeth develop pulp necrosis, apical periodontitis or periapical abscess, some residual vital pulp tissues may exist in the apical region, which can be used in REPs to promote tissue regeneration.^17^ SCAPs were firstly characterized from the apical tissue in 2006,^28^ with capacity of proliferation and odontogenic differentiation that is beneficial for root development.^29–32^ In addition, in combination with the location proximal to teeth apices, SCAPs would be the most promising stem cell source for REPs. PDLSCs and BMSCs are also the potential stem cell sources for REPs as evoked bleeding from the apical tissue may induce the release of these cells.^12,33^ The iPAPCs appear to be largely localized in the vasculature within apical granulomatous tissues, and represent another important potential source of stem cells for REPs.^34^

Studies have proved an increased expression of marrow stem cells (MSCs) markers in the intra-canal blood induced by over-instrumentation into the periapical tissues, both in immature and mature teeth.^35,36^ However, the explicit origin of these MSCs could not be confirmed due to the lack of special markers for the stem cells.

#### Growth factors

Dentin matrix has been considered as a reservoir of growth factors,^37^ which may be released through demineralization of dentin matrix by bacterial acid, irrigation with sodium hypochlorite (NaOCl) and ethylenediaminetetraacetic acid (EDTA), stimulation by calcium hydroxide and silica-calcium biomaterials such as MTA and Biodentine®.^38–40^ Besides, the blood clot formed during REPs also contains certain growth factors.^20^

The dentin-derived growth factors are believed to play a key role in progenitor cell recruitment, proliferation, differentiation, and promoting tissue regeneration.^37,41^ For example, transforming growth factor-β1 (TGF-ß1) and fibroblast growth factor 2 (FGF2) have been implicated in promoting cell migration and proliferation.^33^ Vascular endothelial growth factor (VEGF) plays an important role in cell proliferation and regulation of angiogenesis while bone morphogenetic protein (BMP) and FGF2 mediate the signaling in dentin formation.^33^ Non-collagenous proteins (NCPs) including dentin matrix protein and dentin phosphoprotein may be involved in odontogenesis.^42^

Exogenous growth factors have also been used to produce synergistic effect with autologous growth factors in REPs.^24,43^ Collagen scaffolds loaded with human recombinant platelet‑derived growth factor (rPDGF) have successfully promoted root maturation in an immature tooth with pulp necrosis.^43^ And a clinical trial demonstrated that injectable hydrogel scaffolds impregnated with basic fibroblast growth factor (bFGF) achieved apical healing and continued root development in teeth with pulp necrosis.^44^

#### Scaffolds

A scaffold is a key element for tissue engineering to guide stem cells location and regulate cell proliferation, differentiation or metabolism. It could also help to promote nutrient and gaseous exchanges.^45^ Blood clot, autologous platelet concentrates and synthetic biomaterials could serve as the scaffolds of REPs. Among them, blood clot and autologous platelet concentrates are the most commonly used scaffolds during REPs.

Blood clot has been induced as a scaffold in most of the REPs cases,^43,46–48^ which is a relatively straightforward and simple approach. It allows integrators on cell surfaces to adhere to fibrous components and selectively adsorb cells, supplying growth factors to promote tissue regeneration. However, blood clot is not easy to obtain, and lacks some properties as an ideal scaffold including easy delivery, good mechanical properties, controllable biodegradation, and incorporation of growth factors.^49^ Moreover, the blood clot contains numerous hematopoietic cells, which could release toxic intracellular enzymes during cell death into the microenvironment, compromising stem cell survival.^50^

Another approach for creating a scaffold is the use of autologous platelet concentrates, including platelet‑rich plasma (PRP), platelet-rich fibrin (PRF) and concentrated growth factor (CGF). These autologous scaffolds require minimal manipulation in vitro and are easy to prepare. They consist of three-dimensional fibrin matrix and abundant bioactive molecules, and can degrade over time. Several REPs cases have achieved success with these autologous scaffolds.^51–53^ However, drawbacks are also presented in the clinical use, such as requiring collection of intravenous blood with special equipments and difficult to control the types and concentration of growth factors during preparation. Also, their lack of temporal degradation control and inadequate mechanical strength to support the coronal restoration further compromise their application.^50^

Several exogenous scaffolds including collagen type 1,^43^ hydrogel,^44^ and collagen- hydroxyapatite^54^ have been clinically used in REPs. These scaffolds are usually loaded with growth factors before placing into immature root canals. The resolution of clinical symptom and apical radiographic radiolucency, as well as the continued root development, has indicated its success in clinical application. In addition, decellularized dental pulp has gained some interest in studies as a potential scaffold for pulp regeneration. An in vivo study showed that decellularized dental pulp of swine implanted into pulpectomized teeth in dogs could induce the formation of a vascularized pulp-like tissue with expression of odontoblastic markers.^55^

### Histological evaluation of REPs

Though most case reports and clinical studies have proved increased root length, thickened root wall and decreased size of apical foramen in immature teeth after REPs,^13,20,48,53,56–59^ histological evaluation of the teeth treated with REPs have shown that the newly formed tissue were ectopic bone, cementum, and periodontal ligament instead of dental pulp or pulp-like tissue.^60–63^ Animal studies have also confirmed the above results.^64–66^ Immunohistochemical staining results showed that the expression of osteogenic markers was significantly higher than those in the mineralized tissue,^66^ indicating REPs may promote repair instead of inducing true regeneration. However, Torabinejad et al.^67^ reported the possibility of regeneration of pulp-dentin complex when the apical 1~4 mm of vital pulp remained intact in immature teeth of ferrets. A recent case report has interestingly demonstrated that healthy fibrous connective tissue with blood vessels was found in the canal space and odontoblast-like cells were found along the newly formed dentin wall in the tooth with clinical success for 54 months after REPs.^68^ In addition, the immunohistochemical outcomes showed positive staining of vascular and neuronal markers,^68^ suggesting a partial regeneration of components of the pulp-dentin complex is possible.

## Case selection

### Indications

The REPs is indicated in cases meeting the following criteria: (a) Necrotic permanent teeth with incomplete root formation, regardless of presence of periradicular lesions; (b) No need of post/core for the final restoration; (c) Patients/parents with good compliance; (d) Patients not allergic to medicaments or antibiotics used in the procedures.

The REPs is not applicable in certain cases, such as immediately replanted teeth after avulsion, cases with inadequate tooth isolation, teeth with extensive loss of coronal tissue requiring a post restoration or teeth with endodontic-periodontal lesions.

### Considerations in case selection

#### General condition

General health condition should be firstly considered when making a treatment plan. The American Society of Anesthesiologists’ (ASA) Health Classification System can be used to assess patients’ physical health status.^69^ REPs can be considered for patients who are classified as ASA 1 and ASA 2.

It is difficult to control the infection of root canals in patients with impaired immune diseases such as poorly controlled diabetes or long-term use of hormone drugs. And patients with poorly controlled hypertension, recent (<3 months) myocardial infarction, cerebrovascular accident or coronary artery disease are not recommended to conduct REPs.

Importantly, it should be cautious to conduct REPs in the following cases: (a) Patients with mental health issues, dental phobia or excessive anxiety; (b) Patients can not comply with multiple visits and longer follow-up.^70^

#### Age

Age is another systemic factor taken into consideration for REPs in the reported cases. More than 90% of patients undergoing REPs were below 17 years old.^17^ A clinical study found that better treatment outcome was achieved with greater root development in the younger patient group (9 to 13 years old) compared to the group of 14 to 18 years old,^71^ indicating that REPs can be implemented in any age ranging from 9 to 18 years old, but younger patients (9 to 13 years old) were better candidates for REPs than older ones (14 to 18 years old). Moreover, a few studies reported failure in inducing root development in immature teeth after REPs in patients over 18 years old.^72–75^ This is possibly attributed to a greater healing capacity or stem cell regenerative potential in younger patients, which is evidenced by studies showing that proliferative and differentiation potential of MSCs decreased with aging.^76,77^ Another important factor related to age is the stage of root development. The large diameter of open apex allows the ingrowth of tissue to the root canal space with a rich source of MSCs from the apical papilla. In this regard, the patient’s age appears to be an important factor in case selection. However, further research is required to elucidate the age limit for REPs.

Clinicians should also be aware that REPs is not indicated in deciduous teeth because there would be risk of disturbing the eruption pattern of succeeding permanent teeth.^78^

#### Root morphology

Researches support that root morphology should also be considered in the case selection. Recent studies showed greater increases in root thickness, length, and apical narrowing were found in teeth with preoperatively wider apical diameters (≥1 mm).^71^ Noteworthily, the minimal apical diameter required for REPs is still controversial. Laureys et al.^79^ found newly formed tissue in the root canals with the smallest apical diameter ranging between 0.24 mm and 0.53 mm, while Abada et al.^80^ showed an increase of the in-growth tissue in the root canals with increased apical diameter. And clinical success was achieved in teeth with apical diameter smaller than 1 mm after REPs based on the clinical and radiographic results of periapical healing and continued root development, with the highest clinical success rate of 95.65% in teeth with apical diameters of 0.5~1.0 mm.^78^ Overall, REPs may be applicable in the teeth with apical diameters as small as 0.24 mm or above within the limitation of the current literature. However, no definite conclusion on the correlation between root morphology and REPs outcome has been drawn.

**Fig. 1:**
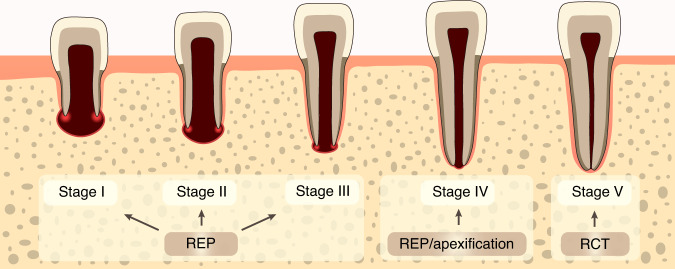
Schematic stages of root development and recommended treatment modalities

As described by Cvek, root development has been classified into 5 stages ((Fig. 1).^81^ REPs has been at the forefront of treatment recommendations for young permanent teeth at stage 1 (less than 1/2 of root formation with open apex), stage 2 (1/2 root formation with open apex) and stage 3 (2/3 of root development with open apex) after developing pulp necrosis. For root development reaching stages 4, both apexification and REPs are recommended as treatment modalities, provided that the thickness and strength of roots are adequate, while RCT is suggested in offending teeth at stage 5. However, REPs has been recently performed on mature teeth (closed apices) with pulp and periapical disease.^26,82^ A clinical study showed that REPs achieved resolution of clinical symptoms and apical radiolucency of various sizes, and half of the teeth after REPs present positive response to electric pulp testing, indicating the treatment potential of REPs in mature teeth with large periapical radiolucency.^83^ However, more evidence-based studies should be implemented before the application of REPs in mature teeth with apical periodontitis.

## Pre-operative preparation

### Patient’s informed consent

For mutual understanding, support and cooperation, the patients, parents or legal guardians should be provided with detailed information bearing on the existing status of the teeth, and the regenerative procedures along with treatment alternatives, such as those below.

- The condition of the affected tooth and its influence on the treatment.
- Treatment time, frequency and follow-up period.
- Material and instruments used for the treatment.
- Therapeutic effect, including the possibility of persistent tooth pain, unhealed or aggravated periapical lesions and post-operative stagnation of the root development.
- Treatment alternatives such as apexification or tooth extraction if necessary.
- Possible complications, such as pain, root canal calcification, tooth discoloration, and options for treating the complications.
- Treatment costs.

Once the information above has been discussed and fully understood, the Informed Consent Form should be signed before starting treatment.

### General health assessment

A thorough assessment of a patient’s health status such as the patient’s medical condition, anesthetic and allergic history, should be completed and properly documented prior to the treatment planning.

Ideally, the information should be gathered during a consultation appointment before the procedures starts, which will help to confirm whether REPs is a proper treatment option. For instance, patients with long-term corticosteroids therapy or severe systemic disease like uncontrolled diabetes mellitus may not be suitable for the regenerative procedures, due to their questionable tolerance in treatment procedures and depressed immunity.^84,85^

Apart from the health status, clear communication should be ensured regarding patient’s experience in previous local and general anesthesia. Even for those who have little or no experience with any form of anesthesia, prevention of adverse reactions should be considered before the treatment. Besides, history of any allergy should be collected and documented in detail. If the patient reports previous allergic reaction to anesthesia, the saftest choice would be mepivacaine or prilocaine without vasopressors, because esters of benzoic acids may cause cross reaction, while this is unlikely among amide local anesthesia.^86^ Furthermore, for patient having atopy whose immune system is more sensitive to common allergic triggers, the antioxidants containing vasopressors and bisulfites should be also avoided.^87^ If the patient is allergic to specific antibiotic, alternative antibiotics or Ca (OH)_2_ should be selected for intracanal medication.

### Oral examination

Before starting treatment, clinical oral examination, pulp sensibility tests, and radiographic examination should be performed to evaluate the amount and configuration of the residual tooth structure, the development of the root and the periodontal conditions of the tooth to confirm whether the infected tooth meets the inclusion or exclusion criteria mentioned above in Case selection part.

## Clinical protocol

**Fig. 2:**
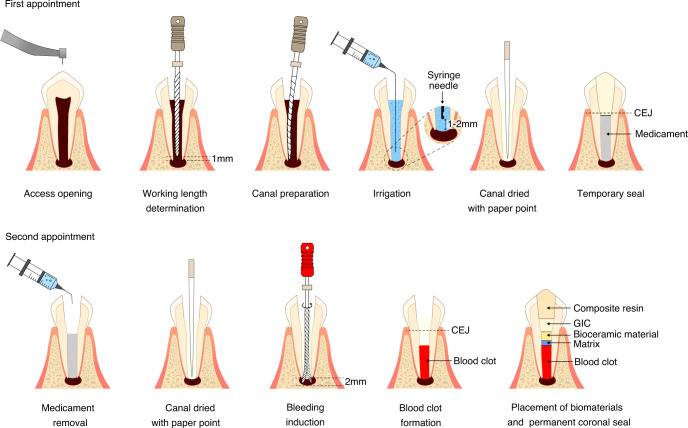
Schematic diagram of REPs

Despite the variations in clinical protocols, the key steps of conducting REPs are (a) Minimal instrumentation of canal wall; (b) Disinfection using irrigants; (c) Dressing with an intracanal medicament; (d) Introducing bleeding into the canal space and creating a blood clot; (e) Capping with bioceramic materials; (f) An effective coronal seal. These issues should be considered when implementing the treatment procedures. The recommended steps of REPs are summarized in Table 1 and Fig. 2 based on the current literature.

**Table 1:** Treatment Procedures of REPs

Appointments		Notes
Procedures of first appointment	Adequate local anesthesia	No emphasis on the use of vasoconstrictors
	Rubber dam isolation	
	Superficial disinfection by 2% chlorhexidine or 2% povidone iodine	
	Removal of coronal infected tissue under dental operating microscope	
	Access cavity preparation	
	Working length determination	By radiograph with a file positioned at 1 mm from apex
	Minimal instrumentation	Using larger size of files or reamers such as Hedström files Circumferentially “brushing” the canal walls without major dentin removal
	Irrigation with 1.5~3% NaOCl (20 mL per canal, 5 min)	Bleeding or exudate may require extended irrigation until they can be controlled with paper points Irrigating needle is suggested to be positioned 1~2 mm from the root end
	Irrigation with sterile saline (5 mL per canal)	
	Dry with paper points	
	Irrigation with 17% EDTA (20 mL per canal, 5 min)	Irrigating needle is suggested to be positioned 1~2 mm from the root end
	Dry with paper points	
	Intra-canal medicament with Ca(OH)_2_ or a low concentration of antibiotic dressing	AAE recommends the use of Ca(OH)_2_ or 1~5 mg·mL^−1^ antibiotic dressing ESE recommends the used of Ca(OH)_2_ The medicament should be placed below the cemento-enamel junction (CEJ)
	Access restoration with temporary restorative material	Cavit™ or glass-ionomer can be used Restoration should be at least 3~4 mm
Procedures of second appointment	Clinical assessment of response to the first treatment	AAE recommends the recall of 1~4 weeks after the first visit ESE recommends the recall of 2~4 weeks after the first visit Repeat the treatment procedures of the first appointment if there are signs or symptoms of persistent infection
	Local anesthesia	3% mepivacaine without vasoconstrictor (epinephrine)
	Rubber dam isolation	
	Superficial disinfection	2% chlorhexidine or 2% povidone iodine is used
	Removal of temporary seal	
	Gentle irrigation with 17% EDTA under microscope to remove the intracanal medicament	AAE has no requirement for the volume and time of irrigation with EDTA ESE suggests the irrigation of 20 mL EDTA in 5 mins followed by 5 mL sterile saline Use ultrasonic activation if necessary
	Dry with paper points	
	Induction of bleeding	By rotating a pre-curved K-file with larger size (for example size #25) at 2 mm past the apical foramen until the whole canal filled with blood below the CEJ level Wait for 15 min for a blood clot formation
	Placement of resorbable matrix over the blood clot	This procedure is optional CollaPlug™, Collacote™ or CollaTape™ can be used The matrix is trimmed into a diameter slightly larger than the coronal part of the root canal
	Placement of tricalcium silicate biomaterial	MTA, Biodentine®, EndoSequence® BC RRM-Fast Set Putty, etc. can be used Capping material should be approximately 2 mm underneath the CEJ
	Application of light-cured glass ionomer	The layer should be at least 3~4 mm over the capping material
	Refreshment of the cavity walls with a diamond bur	
	Permanent restoration	Bonded reinforced composite resin is suggested
Follow-up requirement	The patients should be reviewed at 3 months, 6 months, 12 months, and yearly for a total of 5 years for regular clinical and radiographic examinations.	
	CBCT is highly recommended for initial evaluation and follow-up visits.

### Minimal instrumentation

For immature permanent teeth, the apex diameters usually exceed the diameter of largest files, bringing a challenge for mechanical instrumentation. Moreover, mechanical preparation may further weaken the fragile and thin dentin wall of roots. There is no mechanical instrumentation recommended in the statement of ESE and the most recent guideline of AAE. However, without mechanical instrumentation, bacterial biofilm is likely to remain within dentinal tubules which may lead to a failure in REPs.^88^ Thus instead, minimal instrumentation should be considered in REPs. The canal walls are lightly brushed circumferentially with endodontic instruments such as K files with larger size and Hedström files to disrupt the bacterial biofilms without any aggressive removal of dentin.

### Chemical disinfection

Similar with conventional irrigating protocol in RCT, several requirements of irrigants should be considered in REPs, including antibacterial effect, lower cytotoxicity and stimulation capacity of growth factors release.

The NaOCl is the favorable irrigant in most REPs studies due to its broad antibacterial spectrum and tissue dissolution properties. Although 6% NaOCl has been used in REPs cases and achieved success,^89^ several in vitro studies have shown that NaOCl had a concentration-dependent effect on the survival of SCAPs, with 6% NaOCl significantly reducing the stem cell survival.^90,91^ Thus, a low concentration of NaOCl, is recommended in REPs. On the other hand, 17% EDTA has been proved to rescue the detrimental side effects of NaOCl on stem cells attachment, survival and differentiation,^92^ and promote the release of endogenous growth factors from the dentin.^93^ Based on these evidence, the guidelines of AAE^18^ and ESE^19^ recommend the use of 1.5%~3% NaOCl followed by 17% EDTA in the first appointment and 17% EDTA in the second appointment of REPs. Noteworthily, chlorhexidine is not recommended for REPs because it does not have tissue dissolution capability and has been shown to be cytotoxic to stem cells.^94^

To enhance the efficacy of disinfection in root canals, irrigation methods such as negative pressure irrigation, passive ultrasonic irrigation (PUI), photon-induced photoacoustic streaming (PIPS) or laser, can be used. Negative pressure irrigation demonstrates beneficial effect in minimizing the risk of irrigant extrusion through the apical foramen and has been used in REPs. Needles with closed end and side-vents, or EndoVac™ are suggested irrigation devices, together with copious irrigant with a slow rate of infusion, to reduce the risk of extrusion into the periapical tissue. Importantly, the irrigating needle should be positioned 1~2 mm from apex to minimize cytotoxicity to stem cells seeding in the apical tissues. In addition, PUI can be considered in the second appointment in order to promote tissue dissolution and accelerate growth factors releasing from the dentin walls.^95,96^ An in vitro study demonstrated that irrigation with PIPS activated EDTA for 40 s led to the smear layer removal without undesirable impairment of dentin microhardness. Additionally, this irrigation created more cell-friendly dentin conditioning that was beneficial for SCAPs adhesion and survival.^97^ Divya et al.^98^ showed that incorporating with AAE protocol, use of diode laser in the first appointment contributed to enhanced disinfection and significant outcome of periapical healing in necrotic immature teeth.

### Intra-canal medicaments

A variety of medicaments have been used for root canal disinfection in the REPs, including triple antibiotic paste (TAP) with different combinations, double antibiotic paste (DAP) and Ca (OH)_2_.^24^

The TAP mixed with 1:1:1 ciprofloxacin, metronidazole and minocycline has been reported in 51%~80% of REPs cases,^24^ and its efficacy in disinfecting necrotic root canal systems has been demonstrated.^99,100^ However, the effect of TAP on the survival of SCAPs has also been tested in vitro^101,102^ and shown that high concentrations (10~100 mg·mL^−1^) of TAP were detrimental to the survival of SCAPs. The AAE has suggested a concentration of 1~5 mg·mL^−1^ TAP in REPs to avoid damage of SCAPs.^18^ At this concentration range, the antibiotics are prepared as solution formulation and TAP can be delivered into canal system via a syringe.

It should also be noted that TAP can cause tooth discoloration due to the component of minocycline. DAP without minocycline or replacing minocycline with other antibiotic substitution such as clindamycin and cefaclor are possible alternatives. If TAP is used, a dentin bonding agent can be used to seal the pulp chamber before placing medicament, and TAP is recommended to remain below cement-enamel junction (CEJ) level to minimize crown staining.

Ca (OH)_2_ is another intracanal medicament used in REPs. It appears to be less effective than TAP in antibacterial capacity,^103,104^ but has several advantages over TAP including no discoloration, lower cytotoxicity to stem cells, greater survival and proliferation of stem cells on the treated dentin, promotion of growth factor release from the treated dentin and easier removal from root canals. In addition, Ca (OH)_2_ used in REPs is less likely to reduce fracture resistance because of the relatively short-term use as intracanal dressing for 1~4 weeks. Regarding the above desirable effects, the ESE has recommended Ca (OH)_2_ as the prior intra-medicament in REPs.

Though antibiotic pastes and Ca (OH)_2_ have been used as intracanal medicaments in REPs, there are few studies comparing their effects in REPs. A meta-analysis^105^ presented that antibiotic pastes contributed to a higher percentage of root wall thickening while Ca (OH)_2_ induced a higher percentage of apical closure. Recently, propolis paste has been proposed as an intra-canal medicament and shown similar disinfection effect with TAP in animal studies.^106,107^ And pre-clinical studies found that novel intra-canal drug delivery system using nanofibers appeared to be an alternative of biocompatible disinfection strategy for REPs.^108,109^ Further well-designed high-quality studies are warranted to provide evidence-based recommendation in intra-canal medicament for disinfection.

### Blood clot formation

During REPs, the purpose of inducing intra-canal bleeding is to create a blood clot as a scaffold, and to promote growth factors and stem cells from apical region into canal lumen for tissue regeneration.

In clinical practice, inadequate intra-canal bleeding has been identified as a challenge to successful REPs. Severe destruction of periapical tissues, the resolution of inflammatory reaction after dressing with antibiotic paste and the use of local anesthesia containing epinephrine are the possible reasons for the failure to induce sufficient bleeding.^33,75^ The available guidelines of AAE and ESE have proposed that vasoconstrictor-containing local anesthetics should be avoided at the second appointment to minimize the possibility of inadequate intra-canal bleeding. If bleeding is insufficient, lidocaine, a potent vasodilator without epinephrine could be locally injected before attempting to induce apical bleeding.^110^

PRP, PRF or CGF can also be a clinical alternative in REPs when intra-canal bleeding induction is unsuccessful.^20,111–113^ There are conflicting evidences on their outcomes in REPs. It has been reported PRP or PRF was superior to bleeding induction in promotion of continued root development in REPs.^114^ Furthermore, PRP was better than PRF or induced bleeding in the healing of periapical lesion, while no significant differences with respect to root lengthening and lateral wall thickening.^115^ However, Zhou et al. argued that compared to blood clot alone, the combination of PRF and blood clot did not improve the outcomes of REPs.^60^ Furthermore, there is still lack of evidence to prove that PRP or PRF could improve the regeneration of the pulp-dentin complex. Overall, induced bleeding is more commonly suggested as a scaffold in REPs of a non-vital immature permanent tooth.^115^

## Regenerative endodontic outcomes

### Evaluation of REPs outcomes

There are guidelines from ESE and AAE for evaluating the efficacy of REPs.

The ESE has described a series of success criteria for REPs, including the absence of inflammation, healing of pre-existing bony lesion in the periapical tissue, increased root length and wall thickness, lack of external inflammatory resorption, a positive response to pulp sensibility testing, radiographic detection of new PDL along the inner wall of root canal, and no tooth discoloration.^19^

The AAE categorizes the success criteria of REPs into primary, secondary, and tertiary goals. The primary goal is the elimination of symptoms and bony healing. The secondary goal is the increased root wall thickness and/or root length, which is desirable but not essential. The tertiary goal is the positive response to vitality testing, which could indicate a more organized vital pulp tissue.^18^

These criteria from both guidelines point to an ideal prognosis, involving the clinical manifestations and imaging diagnoses of the teeth, along with the patient’s subjective feelings. However, the targets mentioned above can hardly be achieved concurrently in the clinical scenario nowadays. According to two recent systematic reviews,^116,117^ the goal of resolving the signs/symptoms of infection and periapical healing is generally achievable, with as high chance as 91%~94%. Nevertheless, the increased root length and wall thickness were not always observed, with a wide range of percentage changes of −2.70%~71.43% and −4%~72.67%.^118–120^ The factors causing this lack of response in certain cases still remain undetermined, which might be due to the persistence of bacterial biofilms or antigens, the feature of etiology, and the effect of disinfectants. Besides, the changes in the angulation of film placement might also distort the quantitative assessment of radiographic outcome. In 2017, Linsuwanont et al. claimed that complete root maturation could be observed by conventional two-dimensional radiography whereas defective root development might be identified in CBCT examination in the same case.^121^ The findings raised concerns about the accuracy of evaluation in the published success cases, due to the limitations of using two-dimensional radiographs to interpret three-dimensional structures.

In terms of regain of a positive response to pulp sensibility test after REPs, it was present in 50%~60% of published cases. However, researchers^33,48^ suggested that positive response to pulp sensibility test in immature permanent teeth with pulp necrosis after REPs could not be taken as a proof of pulp regeneration, provided that by now no reorganized vital pulp tissue has been detected in the treated teeth, but just vital tissues containing cementum-like, bone-like tissue and nerve fibers. The vital soft tissues might be able to respond to pulp test to some degree probably because they are vascularized and innervated. In views of the practical limitations and ethical considerations for performing histological studies in humans, it is still a very long journey before we get a definitive answer. Therefore, the classification criterion of AAE might be more appropriate for clinical assessment, which has indeed been used in a variety of published studies.

In 2016, Diogenes et al.^122^ suggested a multilevel approach to evaluate the success of REPs, by three stakeholders: the patients and their legal guardians, clinicians and scientists. Accordingly, they described the REPs outcomes in three levels: patient-centered, clinician-centered and scientist-centered outcomes. Briefly, the patient-centered outcomes include the resolution of infection which is manifested by the absence of swelling, drainage and pain, the functional survival of the teeth, and acceptable esthetics. These outcomes are aligned with patients’ expectations from any given clinical intervention and should be the top priorities for our treatments. From a clinician’s perspective, an ideal REPs is one that presents radiographic signs of healing and root development, along with positive responses to pulp sensibility tests after the treatment. However, no matter what the results will be for the comparative analysis of the treatment effectiveness, the patient-centered outcomes could not be overemphasized. The primary goal is still to promote healing of the diseased tissues and patient well-being. Finally, at the scientist level, the histologic evidence of complete regeneration of the treated teeth is an ultimate goal. However, as the primary stakeholder of their health and of the development of new therapies, patients should be empowered to make decisions about treatment effectiveness, reporting back to clinicians and researchers what has been satisfied or unsatisfied. Overall, this assessment criterion better engages different stakeholders in the treatment outcomes evaluation from different perspectives.

### Causes of treatment failure

It has been reported that REPs has reached a high treatment success rate of 83.3%~100%.^13,123,124^ The main causes of treatment failure are inadequate control of the infection and root resorption after the therapy.

As stated in the inclusion criteria, the teeth receiving REPs are usually young permanent teeth featured with thin canal wall and open apical foramen. Therefore, while conducting the root canal cleaning for REPs, chemical disinfection is always the main focus over mechanical preparation, in order to avoid the excessive removal of dentin from the root canal wall, and minimize the resistance reduction and the risk of root fracture. However, reduced mechanical preparation may leave the bacterial biofilm retained in the dentinal tubules and cause persistent infection in the root canal system.

Another reason for re-infection is the lack of adequate coronal sealing, which not only allows oral microbes to enter the root canal system and cause re-infection, but also impairs the proliferation and differentiation of stem cells, leading to the inhibition of the periapical lesions healing and root development. Other factors such as incomplete removal of tooth caries, detachment of the temporary restoration or tooth fractures between appointments, also compromise the coronal sealing.

Root resorption^125^ was another main reason why REPs was failed. This situation was generally found in trauma cases. Severe trauma to the periodontium would induce inflammatory root resorption and may bring damage to the Hertwig epithelial root sheath as well as the apical papilla, compromising the treatment outcome of regenerative procedures.

## Complications and managements

Common complications in relation to REPs are pain response, tooth discoloration and intracanal calcification. Clinicians should be fully aware of these complications, and how to prevent and manage when they arise (Table 2).

**Table 2:** Complications and managements of REPs

Complications	Etiology	Prevention	Managements
Pain	Mechanical stimulation on periapical tissue	Keep accurate working length during the treatment Operate gently	For severe pain, effective dental drainage should be operated in time and the root canal disinfection should be performed again If the pain continues or aggravates, alternative treatments (apexification or tooth extraction) should be considered
	Compromised anesthesia effect	Use anesthetics with good permeability to improve the pain control	
	Interappointment pain resulting from residual infection within the root canal, or re-infection of the tooth	Isolate tooth strictly by using rubber dam Copious canal irrigation and disinfection Seal the access cavity tightly between appointments	
Tooth discoloration	Minocycline chelates calcium ions and incorporates into the dentin tissue as well as synthesizing oxidation products	Use low concentration of TAP and keep it below CEJ, right after sealing the pulp chamber with a dentin bonding agent Remove or replace minocycline with other antibiotics in TAP, such as clindamycin, amoxicillin and cefaclor	Internal bleaching Invasive intervention such as ceramic restorations could be the treatment of choice
	Blood ingredients permeate into the dentinal tubules and change the refractive index of the teeth	Place resorbable matrix over the blood clot, separating it from the barrier materials	
	Porosities in calcium silicate–based materials entrap blood components and present high staining potential	Modify the composition of barrier materials by reducing the content of bismuth oxide or replacing it with zirconium oxide	
Intra-canal calcification	A compound effect from multiple contributing factors which remains uncertain	–	Further treatment is usually unnecessary unless the tooth is symptomatic If symptoms are evident and show no sign of relief, RCT with guided access, micro-endodontic surgery or even an intentional reimplantation could be the treatment modality In the worst scenarios, tooth extraction could be considered
	Associated with ectopic bone formation and cementogenesis inside the root canals	–	

### Pain

Pain responses may occur during or after REPs. Prevention and management of pain is important to improve patient’s experience in dental treatment. During the treatment, pain response may be caused by mechanical stimulation to the periapical tissue, possibly due to the poor control of the working length when undertaking root canal preparation and irrigation. To avoid this, the working length should be confirmed at the beginning of the treatment and carefully kept during the whole process. Gentle operations should be performed during the root canal irrigation, and the tip of the irrigation needle should be kept at least 1 mm above the periapical tissue to minimize the risk of extending beyond the apical foramen and damaging the periapical tissues. In addition, considering the compromised anesthesia effect by using local anesthetic agents without the decongestant, drugs with good permeability such as 3% mepivacaine can be used to improve the pain control during the treatment. Residual infection within the root canal, or re-infection of the tooth can cause interappointment pain. Therefore, through the whole treatment process, the tooth under treatment should be strictly isolated by rubber dam. Moreover, copious irrigation with NaOCl and EDTA, and placement with intracanal medicament should be applied in order to remove the infection in the canals to the largest extent. The access of the dental pulp cavity must be tightly sealed between appointments to prevent re-infection.

If an inter-appointment emergency or postoperative pain occurs, appropriate management should be conducted after careful inspections to find out the cause. For patients with severe pain, effective dental drainage should be operated in time and the root canal disinfection should be performed again so as to control the infection and ease relevant symptoms. If the pain continues or aggravates, alternative treatments such as the apexification or tooth extraction should be considered according to the individual situation and patient’s desire.

### Tooth discoloration

Literature reported that discoloration of the crown was a common complication of REPs, which may significantly impact patient’s satisfaction and quality of life.^126^ Patients expect both esthetic effect and successful treatment outcomes, especially for the front teeth. Therefore, procedures with possible staining issue must be avoided as much as possible.

Tooth discoloration after the procedures may trace back to the different treatment stages, such as the use of disinfectant pastes, induction of bleeding, or placement of barrier materials.^127^

Intracanal medicaments are used to disinfect the root canals during endodontic treatments. TAP, which contains ciprofloxacin, metronidazole and minocycline, has been used as disinfectant in REPs for over twenty years. As stated previously, the main issue with TAP is causing severe tooth discoloration as it contains minocycline,^128–131^ which can chelate calcium ions and incorporate into the dentin tissue as well as synthesizing oxidation products.^127,132,133^ Berkhoff et al.^134^ reported that TAP could circumferentially present up to 350 μm within the dentin wall, and around 88% of TAP was still left in the root canals, regardless of how vigorous the irrigation process was performed. Therefore, AAE advocated that low concentration of TAP should be used and kept below the CEJ, right after sealing the pulp chamber with a dentin bonding agent in order to minimize the risk of staining. Alternatively, minocycline could be superseded with other antibiotics in TAP, such as clindamycin, amoxicillin and cefaclor.

Induction of periapical bleeding into the canal lumen is a necessary step in REPs. However, the blood clot formed inside the canal could also result in tooth discoloration. Certain blood components, such as hemoglobin, hematin, or erythrocytes, can permeate into the dentinal tubules and then change the refractive index of the teeth, leading to color changes.^135^ Likewise, porosities in calcium silicate–based materials, which are commonly used as coronal barrier materials, may also entrap blood components and present high staining potential.^65,131,136,137^ According to the ESE position statement, to prevent this, a resorbable matrix can be placed over the blood clot, keeping it separated from the barrier materials.^19^

Aside from blood contamination, materials used as coronal barriers are regarded as another factor causing tooth discoloration, owing to their composition, such as iron, aluminum, magnesium oxides and particularly bismuth oxide.^137,138^ Because of the oxidoreductive potential of bismuth oxide, it could be oxidized into bismuth carbonate when in contact with strong oxidizing agents, such as NaOCl or collagen.^138,139^ Black sediment was then produced, and the tooth color changed.^140,141^ Therefore, in recent years, manufacturers have tried to modify the ingredient of bioceramic materials by removing bismuth oxide or replacing it with zirconium oxide, which does not show a high staining potential.^136,138,142^ These new materials, such as Biodentine®, and EndoSequence® BC RRM-Fast Set Putty, have been studied and presented significantly less discoloration compared with MTA.^129,136,137,143^ However, more evidence-based studies are necessary before drawing a more conclusive relationship between the tooth discoloration and the composition of the bioactive materials.

For discolored teeth after REPs, internal bleaching has been shown to be a less invasive treatment option with predictable outcome.^144,145^ If an unsuccessful outcome is obtained, more invasive intervention such as the application of ceramic restorations could be the treatment of choice.

### Intra-canal calcification

Intracanal calcification was also a common issue in teeth treated with REPs,^119,146–148^ and has been defined as revascularization-associated intracanal calcification (RAIC) in 2017 by Song et al.^149^ Researchers identified RAIC in 62.1% of revascularization cases and found the progressive nature of calcification with time. The calcific bodies in root canals were then categorized into two types: calcific barrier (CB) or canal obliteration (CO). The former is mainly precipitated along the mid-root area, leaving the rest of the canal space visible on the radiographs, whereas the latter means complete obliteration of the canal lumen.

To date, the exact physiopathological mechanism of RAIC remains largely unknown. Some researchers claimed that RAIC might result from ectopic bone formation and cementogenesis inside the root canals.^60,62^ As reported, MSCs from different tissue sources would retain their innate differentiative potential that reflects their tissue origin.^132^ Therefore, PDLSCs and BMSCs from alveolar bone, which have been carried into the root canal within the induced blood flow, would settle and differentiate to form cementum/fibrotic tissues and bone matrix, resulting in calcific bodies inside root canals. This hypothesis has been proved by several animal studies, showing ectopic formation of bone, cementum, and fibrotic tissues inside the root canals after REPs.^66,150–152^ However, since some cases without induced bleeding also presented intracanal calcification, RAIC should not be solely a complication attributed to bleeding but likely a compound effect from multiple contributing factors, which remains uncertain. Previous studies have reported that bioactive materials, such as ProRoot MTA and Biodentine, which have been currently used as coronal barriers in REPs, can activate the proliferation and odontogenesis of MSCs and play a role in mineralization.^153–155^ They could significantly stimulate TGF-β1 release from the root dentin, thus promoting mineralization in the canal space.^156^ Moreover, the alkaline environment created by these materials could activate TGF-β1 from a latent form into an active form, which might further enhance cell differentiation and mineralization.^157^ Considering the various types of growth factors present in the dentin matrix, future studies should be designed to investigate the effect of other signaling molecules on excessive mineralization.

Some studies suggested that intracanal calcification should not be considered as a diseased state.^158,159^ However, excessive calcification would possibly complicate alternative endodontic treatments if further infection were to occur, increasing the difficulty in locating the root canal. Consequently, for patients who suffer from RAIC after REPs, further treatment is usually unnecessary unless the tooth is symptomatic. If symptoms are evident and show no sign of relief, RCT with guided access, micro-endodontic surgery or even an intentional reimplantation could be the treatment modality for the tooth. In the worst scenarios, tooth extraction could be considered, followed by a proper rehabilitation procedure.

## Conclusions and expectations

The REPs has presented a favorable outcome in resolution of signs/symptoms and further root development in immature permanent teeth with pulp necrosis. However, unpredictable root maturation of REPs is a major concern raised in the current literature, and evidences suggest that true regeneration of pulp–dentin complex does not occur after REPs. In recent years, the strategies of stem cell transplantation and cell homing are drawing dramatic attention due to their potential to achieve organized dental pulp regeneration.^10^

Stem cell transplantation is a cell-based approach involving the transplantation of exogenous stem cells loaded onto scaffolds into the root canal system to allow regeneration. Pulp/dentin regeneration has been reported in animal studies using exogenously transplanted dental stem cells.^160,161^ In 2017, Nakashima clinically achieved pulp regeneration in permanent teeth with irreversible pulpitis through the transplantation of mobilized dental pulp stem cells (MDPSCs).^162^ In a randomized controlled clinical trial, stem cells from exfoliated deciduous teeth were implanted into immature permanent teeth with pulp necrosis after trauma, and successfully reconstructed three-dimensional pulp tissue with blood vessels and sensory nerves.^163^ These studies indicated that transplantation of stem cells would be a potential approach of whole pulp regeneration. However, this cell-based therapy faces many challenges in clinical translation due to the complexity of procedures, such as pulp extirpation, cell culture, stem/progenitor cell populations sorting, cell expansion, storage and shipping. In addition, potential contamination, difficulty with regulatory approval and the high costs should also be taken into account.

Cell homing has been regarded as cell-free approach to regenerate pulp-dentin complex through chemotaxis of endogenous stem cells into root canal via biological signaling molecules.^164^ Kim et al. has firstly reported regeneration of pulp-like structure via the cell-homing approach in a mouse model. The human pulpless root canals were filled with collagen scaffolds loaded with bFGF, VEGF or PDGF as well as basal NGF and BMP7, and then the canals were transplanted subcutaneously into mice dorsum for 3 weeks. New dentin-like hard tissue and vascular pulp-like tissues with innervation and odontoblast layers were observed under microscope.^165^ Since then, several in vitro studies illustrated the chemotactic effect of different cytokines including stromal cell-derived factor-1α (SDF-1α), stem cell factor (SCF), bFGF and BMP7 on dental stem/progenitor cells to support the cell homing strategy.^166,167^ Yang et al. established an in vivo mice model and found that SDF-1α-loaded scaffolds generated vascularized connective tissues in the canals with fibrous matrix and new dentin.^168^ Cell homing strategies omitted in vitro procedures for stem cell isolation and manipulation. Therefore, it might be simpler and easier to do in clinic as compared to the stem cell transplantation. However, there is a lack of knowledge in the type of growth factors to be used for pulp regeneration.

Overall, rapid advancements have been reported in the field of regenerative endodontics, which have supported and promoted REPs to be performed clinically while the approaches of cell homing and stem cell transplantation are at the preclinical stage. Despite the excellent effects in resolution of apical lesion, the outcomes of pulp regeneration by REPs are still unpredictable. Stem cells transplantation and cell homing are currently proposed as the potential ways to regenerate true pulp tissues with scientific validity. However, prospective clinical trials and histological evaluations are necessary to identify their applications in clinical translation, making them achievable and predictable in dental practice.
